# No evidence of *NRAS* mutation in squamous cell anal carcinoma (SCAC)

**DOI:** 10.1038/srep37621

**Published:** 2016-11-25

**Authors:** Laura Capelli, Andrea Casadei Gardini, Emanuela Scarpi, Giovanni Luca Frassineti, Luca Saragoni, Maurizio Puccetti, Mario Scartozzi, Massimo Giannini, Stefano Tamberi, Jody Corbelli, Paola Ulivi

**Affiliations:** 1 Biosciences Laboratory, Istituto Scientifico Romagnolo per lo Studio e la Cura dei Tumori (IRST) IRCCS, Meldola, Italy; 2 Department of Medical Oncology, Istituto Scientifico Romagnolo per lo Studio e la Cura dei Tumori (IRST) IRCCS, Meldola, Italy; 3 Unit of Biostatistics and Clinical Trials, Istituto Scientifico Romagnolo per lo Studio e la Cura dei Tumori (IRST) IRCCS, Meldola, Italy; 4 Pathology Unit, Morgagni-Pierantoni Hospital, Forlì, Italy; 5 Pathology Unit, S.Maria delle Croci Hospital, Ravenna, Italy; 6 Medical Oncology, University Hospital, University of Cagliari, Cagliari, Italy; 7 Radiotherapy Unit, Macerata Hospital, Macerata, Italy; 8 Medical Oncology Unit, Degli Infermi Hospital, Faenza, Italy

## Abstract

Epidermal growth factor receptor (EGFR) is usually expressed in squamous cell anal carcinoma (SCAC) and anti-EGFR agents could represent a valid treatment strategy, also considering that *KRAS* and *BRAF* mutations are rare events in this type of cancer. However, no data are available on *NRAS* status in SCAC. In this study we analyzed *NRAS* status (exons 2–4) by Pyrosequencing in a case series of 50 SCAC patients previously characterized in our laboratory for *KRAS, BRAF, PIK3CA* mutations and HPV and HIV infections. We found no mutation in *NRAS* gene. These results confirm that since the principal anti-EGFR resistance mechanisms are almost absent in SCAC, anti-EGFR agents should be considered for the treatment of this type of cancer.

Anal cancer accounts for about 1–5% of all gastrointestinal malignancies, and infection of human papilloma virus (HPV) represents the main etiologic factor^1^. The standard treatment for locoregional disease is combined chemoradiation, which is associated with a 3-year disease-free survival of about 70%. Of those patients who relapse following chemoradiotherapy (CRT), around 40% undergo salvage therapy with abdominoperineal resection^2^. Locally advanced or metastatic disease is still incurable, with only limited responses reported for palliative chemotherapy^3^. New therapeutic approaches are required for these patients.

As EGFR expression in anal carcinoma is observed in a high number of patients (about 80–90% of cases)^4^,^5^,^6^, anti-EGFR drugs have been studied for the treatment of SCAC, showing anti-tumoral activity^7^,^8^,^9^,^10^. The use of anti-EGFR monoclonal antibodies (mAb anti-EGFRs) in colorectal cancer has shown that *KRAS* and *NRAS* represent the main resistance mechanisms to this type of treatment and are thus used in clinical practice to select the patients who should not be treated with anti-EGFR therapy^11^. *BRAF* and *PIK3CA* could also have a prognostic role in determining those patients with a worse outcome^12^,^13^,^14^. *KRAS* mutation seems to be a rare event in SCAC. Some studies, including a study from our group, have shown that no *KRAS* mutation is present in SCAC^4^,^5^,^6^,^15^,^16^, whereas others have reported rates of KRAS mutation of 1–5%^17^,^18^. A low frequency of *BRAF* mutations has also been observed in SCAC, with a rate of 0–5%^15^,^17^,^18^. A higher rate of *PIK3CA* mutation between 16% and 22%, has been observed^15^,^18^. However, no correlation with patient prognosis was observed for these mutations, as well as for HPV infection. To date, no data are available on the frequency of *NRAS* mutation in SCAC. In this study we analyzed the status of *NRAS* in a case series of SCAC previously characterized for HPV, *KRAS, BRAF* and *PIK3CA*.

## Materials and Methods

### Case Series

We analyzed a case series of 50 consecutive SCAC patients treated with chemotherapy and radiotherapy at Istituto Scientifico Romagnolo per lo Studio e la Cura dei Tumori (IRST) IRCCS in Meldola and at the Medical Oncology Units of Faenza, Ravenna and Macerata Hospitals from March 2001 to August 2012, for whom histological material was available. Forty-eight patients received the Nigro scheme with standard dosages^19^, while the remaining 2 patients underwent treatment with cisplatin plus 5-fluorouracil. This case series was previously characterized by our group for HPV infection, *KRAS* (exons 2–4), *BRAF* (exons 11 and 15) and *PIK3CA* (exons 9 and 20) status^15^.

The study protocol was reviewed and approved by the Medical Scientific Committee of IRST IRCCS, and written informed consent was obtained from patients or from their next of kin for the use of biological samples for research purposes. In addition, the experiments in this study were conducted in accordance with approved guidelines and regulations.

### Mutation analysis

Genomic DNA was extracted from Formalin-fixed paraffin-embedded (FFPE) tumor blocks, as previously described^15^. *NRAS* (exons 2, 3 and 4) status was analyzed by Pyrosequencing using anti-EGFR MoAb response (*NRAS* status) (Diatech, Jesi, Ancona, Italy), according to the manufacturer’s instructions. Reactions were run on a PyroMark Q96 ID (Qiagen, Hilden, Germany).

### Statistical analyses

Descriptive statistics were reported as frequencies and percentages for categorical variables and median and range for continuous variables. Progression-free survival (PFS) was calculated from the first day of treatment to the date of first observation of disease progression or death resulting from any cause, whichever occurred first, or the last follow-up for patients who were still alive and had not progressed. Overall survival (OS) was calculated from the first day of treatment to the date of death from any cause or the last follow-up. PFS, OS and their 95% confidence intervals (95% CI) were estimated using the Kaplan-Meier life-table method and survival curves were compared by the logrank test. Statistical significance was assumed for P < 0.05. Statistical analyses were carried out with SAS Statistical software (version 9.4, SAS Institute, Cary, NC, USA).

## Results

Clinical-pathological characteristics of SCAC patients included in this study are reported in Table 1. Fifty patients (19 males, 31 females) were analyzed. Median age was 62 years (range, 37–87 years). Twenty-nine (58%) patients had early stage disease (stage I and II), 19 (38%) had regional nodal involvement (stage III), and 2 (4%) had distant metastasis (stage IV). All patients were *KRAS* (exons 2–4) and *BRAF* (exons 11 and 15) wild-type (wt), whereas 11 showed a *PIK3CA* mutation (8 at exon 9 and 3 at exon 20). All patients had HPV infection (45 HPV type 16 and 5 other HPV types), 6 of which had also HIV infection. All 50 patients resulted wt for codons 12–13 (exon 2), 58-59-61 (exon 3) and 117–146 (exon 4) of *NRAS* gene.

**Figure 1:**
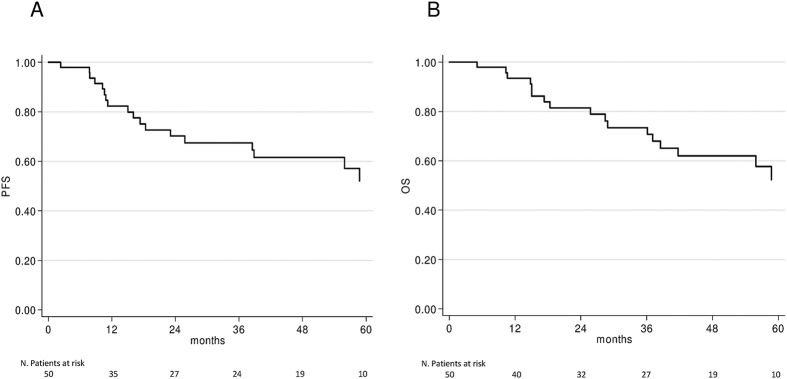
Kaplan-Meier survival curves of (**A**) progression-free survival (PFS) and (**B**) overall survival (OS).

At a median follow-up of 50 months (range 2 to 178 months), 11 patients had progressed (7 with local and 4 with distant relapse) and 10 had died from causes other than SCAC. PFS was 82% (95% CI 71–93) at one year, 67% (95% CI 53–82) at 3 years, and 52% (95% CI 34–70) at 5 years. OS was 93% (95% CI 86–100) at 1 year, 73% (95% CI 60–87) at 3 years, and 52% (95% CI 34–70) at 5 years. (Fig. 1 and Table 2). PFS and OS in relation to clinical-pathological characteristics of patients and in accordance to *PIK3CA* status, HIV and HPV infections are reported in Table 2. Tumor grade and size, lymph node involvement and stage were significantly associated with PFS and OS. Presence of HIV infection, type of HPV infection and *PIK3CA* status were not correlated with survival.

## Discussion

The aim of our study was to analyze the status of *NRAS* gene in a case series of SCAC patients previously characterized in our laboratory for *KRAS, BRAF* and *PIK3CA* genes. To our knowledge, this is the first study to analyze *NRAS* status in SCAC. Given that mutations occurring on *KRAS* exon 2–4 in colorectal cancer – in addition to those occurring on *NRAS* exon 2–4 – may play a role in determining resistance to moAb-EGFRs^11^, these molecular characterizations could also be investigated in SCAC in view of the potential usefulness of these antibodies in this type of cancer.

Other studies have analyzed the frequency of *KRAS, BRAF* and *PIK3CA* mutations in SCAC, showing a very low rate of *KRAS* and *BRAF* mutations^15^,^16^,^17^,^18^, and a slightly higher frequency of *PIK3CA* mutation^15^,^18^. No correlation between *PIK3CA* mutation and patients prognosis was reported in this study with an updated follow up, confirming what previously stated^15^.

We demonstrated that no *NRAS* mutation is present in SCAC and confirmed the absence of potential resistance mechanisms to moAb-EGFR, such as cetuximab or panitumumab. This data, together with previous evidence on *KRAS* and *BRAF*, suggest that cetuximab could be a valid treatment strategy in SCAC.

We confirmed that *PIK3CA* mutations have no prognostic role in this type of cancer, whereas tumor grade and size, lymph node involvement and stage are significantly associated with PFS and OS. Moreover, infection of HPV 16 was significantly associated with a longer PFS but not with OS.

A recent phase I study has demonstrated that the adding of cetuximab to a chemo-radiotherapy with 5-fluorouracil (5-FU) and mitomycin-C in SCAC induced a complete remission rate of 73% with a relative high toxicity^10^. High toxicity was observed also in other clinical trials^20^,^21^, suggesting that the relation between toxicity, chemotherapy dosage and radiation techniques should be better clarified to optimize this promising combination treatment strategy for SCAC. In addition to this data, a recent case report has shown a dramatic response to cetuximab in combination with cisplatin and 5-FU in a patient with metastatic anal cancer^9^.

Moreover, there are no therapies available after progression to cisplatin and 5-FU that can improve survival. A case report showed a complete response to cetuximab in monochemotherapy in a patient with refractory metastatic anal carcinoma suggesting a possible use in this setting^22^.

There are numerous similarities between SCAC and head and neck squamous cell carcinoma (HNSCC), both in terms of etiology and pathology. In particular, as observed in SCAC, high EGFR expression and a low percentage of RAS mutations have also been reported in HNSCC^23^,^24^,^25^, and this was the basic starting point for research into mAb anti-EGFRs in this type of cancer. The approval of cetuximab for the treatment of advanced and metastatic HNSCC^26^,^27^ further reinforces the potential effectiveness of anti-EGFR treatment in SCAC.

A limitation of the study consists in the fact that, due to its retrospective nature, not all information on the chemoradiotherapy administered was available, in particular that pertaining to the different radiotherapy dosages delivered. Moreover, despite the known disadvantages of a composite endpoint, we decided to use such an endpoint because of the potential underreporting of disease recurrences caused by the non-homogeneous availability of follow-up data from the different centers involved in the study.

Our results demonstrated that *NRAS* gene is wt in SCAC patients. These data, together with those reported on *KRAS* status, seem to confirm the potential usefulness of anti-EGFR drugs in the treatment of SCAC. Results of ongoing clinical trials will clarify the tolerability and the efficacy of these agents in combination with conventional CRT.

## Additional Information

**How to cite this article**: Capelli, L. *et al*. No evidence of *NRAS* mutation in squamous cell anal carcinoma (SCAC). *Sci. Rep.* **6**, 37621; doi: 10.1038/srep37621 (2016).

**Publisher's note:** Springer Nature remains neutral with regard to jurisdictional claims in published maps and institutional affiliations.

## Figures and Tables

**Table 1:** Clinical-pathological characteristics of patients.

	*n* (%)
Gender	
Female	31 (62)
Male	19 (38)
Age, years	
<50	7 (14)
≥50 and <70	29 (58)
≥70	14 (28)
Grade	
G1	2 (4)
G2	32 (64)
G3	16 (32)
TNM (T)	
t1-2	27 (54)
t3-4	23 (46)
Stage	
I	8 (16)
II	21 (42)
III	19 (38)
IV	2 (4)
HIV infection	
Yes	6 (12)
No	44 (88)
HPV infection	
HPV-16	45 (90)
Other HPV	5 (10)
PIK3CA status	
wt	39 (78)
mutated	11 (22)
KRAS status	
wt	50 (100)
mutated	—
BRAF status	
wt	50 (100)
mutated	—

HIV, human immunodeficiency virus; HPV, human papillomavirus.

wt, wild type.

**Table 2:** PFS and OS in relation to clinical-pathological characteristics of patients.

	PFS					OS			
	*n* patients	% 1 year (95% CI)	% 3 years (95% CI)	% 5 years (95% CI)	*P*	% 1 year (95% CI)	% 3 years (95% CI)	% 5 years (95% CI)	*P*
Overall	50	82 (71–93)	67 (53–82)	52 (34–70)	—	93 (86–100)	73 (60–87)	52 (34–70)	—
Grade									
1 + 2	34	87 (75–99)	76 (60–92)	55 (31–79)		93 (85–100)	78 (63–94)	59 (36–82)	
3	16	73 (51–96)	49 (22–76)	39 (11–67)	0.048	93 (81–100)	63 (37–89)	36 (8–63)	0.040
T									
1–2	27	92 (81–100)	87 (73–100)	58 (32–84)		96 (88–100)	86 (72–100)	62 (35–88)	
3–4	23	76 (55–97)	46 (19–73)	46 (19–73)	0.020	88 (73–100)	53 (26–79)	44 (17–71)	0.014
Lymph node status									
Negative	31	86 (73–99)	78 (63–94)	57 (35–79)		100	85 (71–99)	59 (37–81)	
Positive	19	76 (56–97)	44 (17–72)	44 (17–42)	0.037	82 (63–100)	51 (24–79)	41 (13–69)	0.013
Stage									
1	8	100	100	83 (54–100)		100	100	83 (54–100)	
2	21	85 (70–100)	80 (63–98)	56 (30–83)		100	78 (59–97)	62 (36–87)	
3	19	76 (55–96)	43 (15–71)	43 (15–71)		87 (71–100)	65 (39–90)	37 (9–65)	
4	2	50 (0–100)	0	0	0.0003	50 (0–100)	0	0	<0.0001
HIV status									
Negative	44	80 (68–92)	69 (55–84)	53 (35–72)		93 (85–100)	74 (60–88)	55 (37–73)	
Positive	6	100	33 (0–87)	33 (0–87)	0.521	100	67 (13–100)	0	0.324
PIK3CA status									
wt	39	83 (71–96)	68 (52–84)	54 (34–73)		92 (83–100)	73 (57–88)	56 (37–75)	
mutated	11	78 (51–100)	65 (32–97)	43 (2–84)	0.883	100	75 (45–100)	30 (0–75)	0.860
HPV									
16	45	80 (68–92)	66 (51–81)	51 (32–71)		93 (85–100)	73 (58–87)	48 (28–68)	
#16	5	100	80 (45–100)	53 (5–100)	0.904	100	80 (45–100)	80 (45–100)	0.854

PFS, progression-free survival; OS, overall survival; HIV, human immunodeficiency virus; HPV, human papillomavirus; wt, wild type.
